# Theoretical Model of the Relationship between Single Embryo Transfer Rate and Multiple Pregnancy Rate in Japan

**DOI:** 10.1155/2012/620753

**Published:** 2012-07-30

**Authors:** Syuichi Ooki

**Affiliations:** Department of Health Science, Ishikawa Prefectural Nursing University, 1-1, Gakuendai, Kahoku 929-1210, Japan

## Abstract

The purpose of the present study was to examine the effect of single embryo transfer (SET) in assisted reproductive technology (ART) on the reduction of the multiple pregnancy rate. We also estimated the monozygotic (MZ) twinning rates according to the SET diffusion indirectly. A reverse sigmoid curve was assumed and examined using nationwide data of SET from 2007 to 2009 in Japan. The multiple pregnancy rate decreased almost linearly where the SET pregnancy rate was between about 40% and 80% of regression approximation. The linear approximation overestimated multiple pregnancy rates in an early period and underestimated multiple pregnancy rates in the final period. The multiple pregnancy rate seemed to be influenced by the improvement of the total pregnancy rate of ART in the early period and by the MZ twinning after SET in the final period. The estimated MZ twinning rate after SET was around 2%.

## 1. Introduction

As is well known, multiple births occur far more often in assisted reproductive technology (ART) than spontaneous conception in almost all developed countries [1–8]. The increase in multiple births is one of the most serious problems related to fertility treatment or ART since multiple births are well known to carry a higher risk of perinatal mortality, preterm birth, and cerebral palsy, and many public health issues resulting from multiple births have become obvious [6].

The multiple birth rate (per 1,000 live births) increased twice during the past two decades in Japan [6]. This rapid increase in the observed number of multiple births and the multiple birth rate is mainly due to iatrogenic, not spontaneous, multiple births of advanced age mothers, especially in the 30- to 34-year-old group [8]. According to the Japanese ART and vital statistics, the percentage of ART live births increased linearly from 0.22% (2,626/1,208,989) in 1992 to 1.64% (18,168/1,110,721) in 2004 to 2.49% (26,680/1,070,035) in 2009. Thus, the use of ART is becoming widespread in Japan.

Single embryo transfer (SET), recently more specifically elective SET (eSET), has been recommended to reduce multiple pregnancy in ART [9–12], and many developed countries have adopted this practice, although it is not known exactly when the use of SET began to spread. The effectiveness and technique of SET have been improved in recent years. Many findings have been accumulated that demonstrate that the use of SET dramatically decreases the twinning rate without lowering pregnancy rates [13, 14]. The guideline of the Japan Society of Obstetrics and Gynecology (JSOG) for ART in 1996 stated that embryo transfer should be limited to three, while the 2008 guidelines specified SET in principle. The rapid increase of ART multiple births after the late 1980s slowed between 1994 and 2005 and rapidly decreased after 2005, especially that of triplets/+ [7]. The secular trend of ART multiples probably reflects the changes in the JSOG guidelines.

There are many studies that have examined the effect of SET on the multiple pregnancy rate [9–14]; however, most results have been based on cross-sectional single- and multiyear data. The purposes of the present study were to examine the theoretical aspect of the effect of SET on the reduction of the multiple pregnancy rate and to estimate the MZ twinning rate according to the SET diffusion indirectly by using nationwide data of ART.

## 2. Methods

### 2.1. Definitions

Pregnancy is defined as ascertainment of a gestational sac and not merely positive reaction to a pregnancy test. Ectopic pregnancies were included. The pregnancy rate after ART was defined as the total number of pregnancies divided by the total number of times of implantation. The SET pregnancy rate was defined as the proportion of the number of pregnancies after SET divided by the total number of pregnancies after ART. In the present definition, the SET pregnancy rate was not the pregnancy rate by SET. The multiple pregnancy rate was defined as the proportion of the total number of multiple pregnancies after ART divided by the total number of pregnancies after ART. The non-SET, mainly double embryo transfer (DET), multiple pregnancy rate was defined as the proportion of the total number of multiple pregnancies after ART divided by the total number of non-SET pregnancies after ART, which was calculated as (total number of multiple pregnancies)/(total number of pregnancies−total number of SET pregnancies). All rates are shown as percentages in the results.

### 2.2. Theoretical Model

The following is a theoretical model of the relationship between the SET pregnancy rate and the multiple pregnancy rate. In a certain year, the total multiple pregnancy rate (*M*) is the sum of the multiple pregnancy rate after SET (*S*) and that after non-SET (*D*), namely, more than one embryo transfer. If the SET pregnancy rate is *s* (0 ≤ *s* ≤ 1), then the non-SET pregnancy rate is 1 − *s*. In addition, if the multiple pregnancy rate after SET pregnancies is *p* (0 < *p*) and that after non-SET pregnancies is *q* (0 < *q*), the total multiple pregnancy rate is calculated by the following formula:

(1) M=S+D=s∗p+(1−s)∗q=q−(q−p)∗s,

where ∗ means multiplication.

All multiple pregnancies after SET produce monozygotic (MZ) multiples, mainly MZ twins, and those after non-SET mainly produce polyzygotic multiples, that is, double embryo transfers produce dizygotic twins. Remember, however, that MZ twinning can occur even after non-SET and thus *q* also contains a portion of MZ twinning after non-SET.

In the present study the relationship between the SET pregnancy rate and the multiple pregnancy rate was treated as the survival curve of the multiple pregnancy rate (*Y*-axis) against the SET pregnancy rate (*X*-axis). To examine this analysis, we assumed that *p* and *q* changed according to the improvement of ART prognosis, the proxy variable of which would be the total pregnancy rate per implantation. Even if *p* and *q* both change, it is reasonable to assume that *q* is larger than *p*, and thus the survival curve would decrease monotonically. The survival curve of the multiple pregnancy rate against the SET pregnancy rate was hypothesized to be divided into the following three periods. (1) First period: when the pregnancy rate per implantation is increasing from a relatively lower level to a higher level, the effect of SET on the multiple pregnancy rate would be small, since multiple embryo transfer does not necessarily produce a multiple pregnancy. The MZ twinning rate after SET (*p*) would decrease and the multiple pregnancy rate after non-SET (*q*) increase according to the improvement of ART, and thus *q* − *p* increases in an earlier stage. This means that the theoretical survival curve has a convex upward shape in this period. The multiple pregnancy rate decreases gradually. (2) Second period: when the pregnancy rate per implantation is nearly constant at a high level, the effect of SET on multiple pregnancies rapidly becomes large. According to the above theoretical formula, if both *p* and *q* are nearly constant or change within a narrow range, the multiple pregnancy rate decreases linearly with the increase of the *s*. The multiple pregnancy rate after SET is much lower than that after non-SET, and thus *q* − *p* is a plus quantity and is nearly equal to *q*. (3) Third period: the pregnancy rate per implantation becomes constant or possibly decreases while remaining at a relatively high level since high-risk-fertility couples do not necessarily achieve pregnancy even with improved ART. In this period, the effect of MZ twinning after SET on the total multiple pregnancy rate would not be ignorable. The multiple pregnancy rate after non-SET (*q*) may decrease, and thus *q* − *p* would also decrease, assuming that *p* is nearly constant. As a result, the decrease of the multiple pregnancy rate became slow, and the survival curve shows a convex downward shape. As mentioned above, the total survival curve of these three periods is expected to be like a reverse sigmoid curve. Remember that this curve depicts the multiple pregnancy rate against the SET rate, not the calendar year, since the SET rate does not increase constantly against the calendar year.

### 2.3. Statistical Methods

The relationship between the SET pregnancy rate and the multiple pregnancy rate was examined by using limited Japanese national data on ART. Almost all medical institutions performing ART are registered with the JSOG [8], which administers questionnaire surveys for these medical institutions. Some of the survey data are presented in simple annual reports of aggregate, not individual, data (in Japanese). Reliable data on the total pregnancy rate per implantation and the multiple pregnancy rate from 1992 to 2009 (the latest) are available. The information on the SET was added from 2007 to 2009. The proportion of eSET among total SET, however, was not reported. The mean response rate for ART surveillance between 2007 and 2009 was 99.3% (1,828/1,840), meaning it is almost all of the data reflecting the current situation of SET and multiple pregnancy in Japan. 

First, we calculated the secular trend between the total pregnancy rate and the multiple pregnancy rate. Then, we calculated the SET pregnancy rate, multiple pregnancy rate, non-SET pregnancy rate, and total pregnancy rate according to the type of ART method employed, including in vitro fertilization and embryo transfer (IVF-ET) and intracytoplasmic sperm injection (ICSI) using fresh embryo/egg, and treatment using frozen embryo, which were the published classification forms in the JSOG annual reports. Subtotal and total pregnancy rates included other methods represented by small numbers, such as gamete intrafallopian transfer (GIFT). Finally, the relationship between the SET pregnancy rate and the multiple pregnancy rate from 2007 to 2009 was plotted, and linear, quadratic, and exponential approximations were performed. The quadratic approximation of three points theoretically produced a perfect fit (*R* ^2^ = 1).

## 3. Results

**Figure 1:**
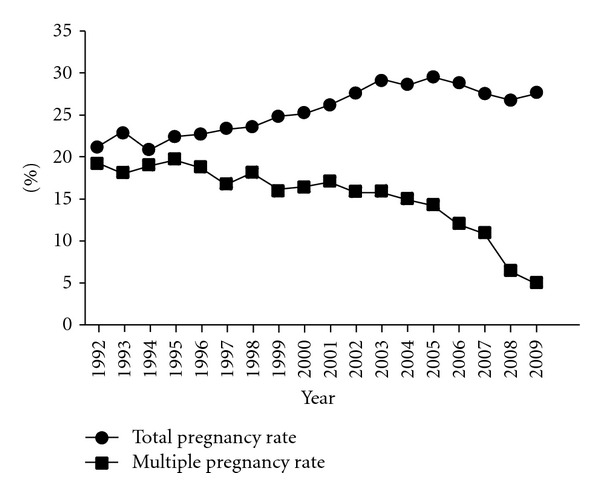
Secular trend of total pregnancy rate and multiple pregnancy rate, 1992–2009.

The secular trend of the total pregnancy rate and multiple pregnancy rate after ART is shown in Figure 1. The total pregnancy rate was nearly constant from 1992 to 1998 (21–23%). It then gradually increased and then tended to decrease from 2005. The multiple pregnancy rate was nearly constant (18–20%) from 1992 to 1996. It then gradually decreased and rapidly decreased from 2007.

The SET pregnancy rate, multiple pregnancy rate, and non-SET multiple pregnancy rate are shown with the total pregnancy rate according to the main methods of ART in Table 1. The SET pregnancy rate rapidly increased during 2007–2009, reaching about 70%, while the multiple pregnancy rate decreased to less than 10%, with no dramatic change in the total pregnancy rate. The non-SET multiple pregnancy rate was nearly constant (19–21% in total) irrespective of the ART method. This value was near that of the multiple pregnancy rate from 1992 to 1996. The SET pregnancy rate was higher in cases of frozen embryo transfer compared to IVF-ET and ICSI.

**Figure 2:**
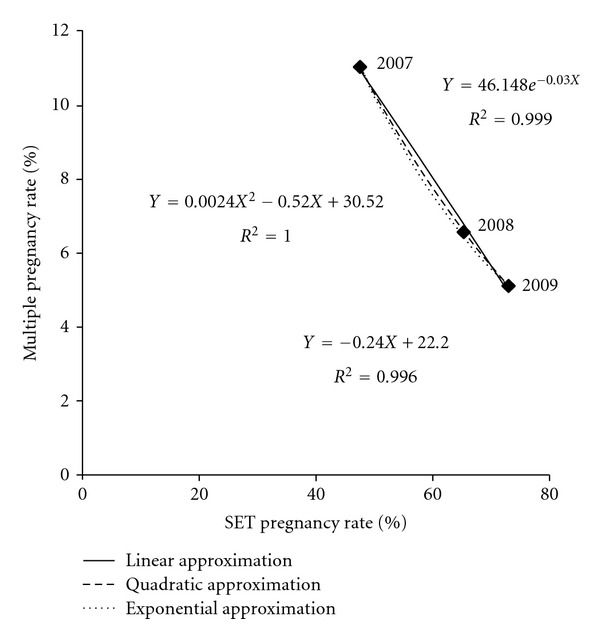
The multiple pregnancy rate plotted against SET pregnancy rate from 2007 to 2009 with linear and quadratic approximation.

The total multiple pregnancy rate plotted against the SET pregnancy rate is shown in Figure 2. The scatterplot tended to decrease linearly. Thus, the regression line was reasonably linear. With this result, the following approximation was performed.

Linear, quadratic, and exponential approximation formulae with corresponding *R* ^2^ *s* according to the ART methods are shown in Table 2. The multiple pregnancy rate when the SET pregnancy rate is 100%, which means MZ multiple pregnancy rate after SET, in approximation formula is also shown. The multiple pregnancy rates all decreased linearly with the increase of the SET pregnancy rate, irrespective of the ART method. All *R* ^2^ *s* were more than 0.99. The regression coefficients of linear approximation were around −0.24-−0.25, irrespective of the ART method. When the SET rate was equal to 0%, the *Y*-intercept of the regression line was 21–23%. Exponential approximations also fit very well. When the SET rate was equal to 100%, the multiple pregnancy rate was estimated to be about 2% in total, showing MZ multiple pregnancy rate after SET. The effect of SET on the decrease of multiple pregnancy, which was shown by the quadratic coefficient of a quadratic curve, was more obvious in frozen embryo transfer compared to IVF-ET or ICSI. These results were not changed, if all analyses were performed by excluding ectopic pregnancies.

## 4. Discussion

### 4.1. Theoretical Model

This study may be the first theoretical examination of the effect of SET diffusion on the prevention of multiple pregnancy. Given the limitation of available data, the estimation method itself need not necessarily be discussed in detail. The present results overall suggested that the theoretical model of a reverse sigmoid curve or similar pattern curve, although most parts were close to a straight line, fit well for the multiple pregnancy rate against the SET pregnancy rate.

The multiple pregnancy rate decreased almost linearly at least when the SET pregnancy rate was between 40% (IVF-ET and ICSI in 2007) and 80% (frozen embryo transfer in 2009), as shown in Tables 1 and 2. This also meant that multiple pregnancy rates decreased linearly at least from 13% to 5%, correspondingly. According to the linear approximation, the regression coefficient was constantly near −0.24-−0.25, irrespective of the ART method, suggesting that *q* − *p* (the non-SET multiple pregnancy rate minus the SET multiple pregnancy rate) in the theoretical formula was near constant. If the SET multiple pregnancy rate is estimated to be around 2%, as mentioned later, then the non-SET multiple pregnancy rate is around 26% (if *q* − *p* = 24% and *p* = 2% then *q* = 26%). This value is slightly lower than the recent total pregnancy rate shown in Figure 1, but it is clearly higher than the expected pregnancy rate of independent two embryo transfer, meaning the DET procedure results in many more instances of multiple pregnancy than singleton pregnancy due to one-embryo abortion.

The linear approximation overestimated multiple pregnancy rates in an earlier period. The multiple pregnancy rate from 1992 to 1997 (18–20%) and the estimated non-SET multiple pregnancy rate (19–21%) were nearly consistent, and they were slightly lower than the *Y*-intercept of linear approximation (21–23%). If this value is reliable as the non-SET multiple pregnancy rate, the effect of SET on the decrease of multiple pregnancy may be gradual.

There have been few studies that analyzed the secular trend of the SET rate and multiple pregnancy rate. Among them, De Sutter et al. [15] found that eSET increased from 1.5% (1997-1998) to 17.5% (1999–2002) of all transfers. Comparing these two periods, an overall pregnancy rate of 35% and 34% per transfer, respectively, was obtained, while the overall twinning rate dropped from 30% to 21%. De Sutter et al. [15] concluded that a decline in the twinning rate is feasible without a drop in the overall pregnancy rate. If this tendency is applicable to the present study, the SET rate was very low in 1997, and most multiple pregnancy rates are based on non-SET. This result also supports that the non-SET multiple pregnancy rate in Japan is around 20%.

On the other hand, the linear approximation was underestimated in the later period. The regression line showed that the multiple pregnancy rate reached zero before the SET pregnancy rate became 100%, suggesting a nonlinear decrease of the multiple pregnancy rate when the SET pregnancy rate reached a certain degree. Moreover, all quadratic or exponential approximation curves were convex in the downward direction, also suggesting recent slowing of the decrease in the multiple pregnancy rate.

### 4.2. MZ Multiple Pregnancies after SET

It is well established that MZ twin pregnancy increased after the introduction of ART [16–18]. According to the recent systematic review and meta-analysis by Vitthala et al. [19], the risk of MZ twinning pregnancy/birth in all ART is 0.9%, and it is a 2.25 times higher than in the case of natural conception. Only three studies [18, 20, 21] were reported on the incidence of MZ twinning after SET in this systematic review. In these three studies there existed 38 MZ pregnancies in a total of 1,850 pregnancies, namely, an MZ twinning rate of 2.05%. According to the present quadratic approximation, the multiple pregnancy rate after 100% of SET was 2.03%, which was in very good accordance with the above results. Recent studies [22–24] also reported consistently MZ twinning rates of 1.9%–2.2%, whether SET occurred or not. The modeling estimation of the twin live birth rate of eSET showed 2.5% for 32-year-old women, 2.3% for 36-year-old women, and 1.9% for 39-year-old women, respectively [12].

Recently, MZ twinning after blastocyst transfer was reported to be significantly higher compared with cleavage-stage embryo transfer [25, 26], although some studies did not support this finding [24, 27]. A recent systematic review and meta-analysis by Chang et al. [28] showed that the risk of MZ twinning after blastocyst transfer was significantly higher compared with cleavage-stage embryo transfer (odds ratio 3.04). Thus, MZ twinning after ART was associated with prolonged embryo culture and should be evaluated considering the stage of the embryo. According to the study of Moayeri et al. [27], the risk of MZ twinning with blastocyst culture is significantly lower recently because of the improvement in culture conditions and a larger experience with blastocyst culture. This supports the present assumption that *p* (the MZ twinning rate after SET) decreased with the advance of ART. Sunde [29] reported that an eSET policy was started in 2002 in Norway and that SET is performed more than 90% as often as IVF or ICSI and the multiple pregnancy rate is well below 10%. These results suggest that it is important to examine secular trends and methods of ART, both likely to be confounded, to estimate the MZ twinning rate after ART.

### 4.3. SET Policy

The main target of SET is twin-prone younger women [30], a group in whom SET is very effective. Preventing ART twins from the remaining groups of patients who attempt it constitutes another and probably tougher challenge, because the overall target group is a heterogeneous mix of patients in very different clinical situations [30]. This means that 100% of SET implementation is virtually impossible. In fact, the Japanese guideline states that embryo transfer should be limited to one (single embryo transfer) in principle but that double embryo transfer is permitted for those women who, for example, are aged 35 or more, and who have failed to become pregnant after ART more than two times successively. 

Recently, Scotland et al. [12] performed an excellent modeling study in which they assessed the costs, consequences, and cost-utility of eSET versus DET. According to their results, eSET is likely to be the preferred option for most woman aged ≤36 years and the decision may best be considered on a case-by-case basis for woman aged 37–39 years. Thus, determining the effectiveness of SET requires the examination of many complicated factors, for example, clinical setting of patients, total pregnancy rate, cost-effectiveness, including patients' quality of life. This is beyond the purpose of the present research.

The multiple births rate could not be analyzed in the present study because the data was insufficient. The multiple pregnancy rate is important from a biomedical point of view, while the multiple births rate after ART is more important from social and public health points of view.

## 5. Conclusions

To conclude, the present study for the first time examined the theoretical aspects of the relationship between the SET rate and the multiple pregnancy rate, which seemed near the reverse sigmoid curve, with almost linear reduction of the multiple pregnancy rate in the period without a large decrease of total pregnancy rate. The estimated MZ twinning rate after SET was around 2%. These results are useful for the evaluation of the total effects of the SET policy on fertility treatment.

## Figures and Tables

**Table 1:** SET pregnancy rate, multiple pregnancy rate, non-SET multiple pregnancy rate, and total pregnancy rate according to the ART type.

			SET pregnancy rate	Multiple pregnancy rate	Non-SET multiple pregnancy rate	Total pregnancy rate per implantation
		2007	41.3 (3,017/7,313)	12.7 (926/7,313)	21.6 (926/4,296)	26.4 (7,313/27,729)
	IVF-ET	2008	60.0 (4,082/6,808)	7.5 (513/6,808)	18.8 (513/2,726)	23.8 (6,808/28,609)
Fresh		2009	69.3 (4,725/6,818)	5.8 (397/6,818)	19.0 (397/2,093)	24.3 (6,818/28,075)
		2007	39.3 (2,585/6,577)	11.3 (746/6,577)	18.7 (746/3,992)	22.1 (6,577/29,768)
	ICSI	2008	55.8 (3,314/5,934)	7.2 (425/5,934)	16.2 (425/2,620)	19.9 (5,934/29,831)
		2009	63.4 (3,921/6,186)	5.7 (354/6,186)	15.6 (354/2,265)	20.2 (6,186/30,604)

		2007	55.7 (7,757/13,932)	9.9 (1,376/13,932)	22.3 (1,376/6,175)	32.1 (13,932/43,452)
Frozen		2008	71.0 (12,913/18,194)	6.0 (1,086/18,194)	20.6 (1,086/5,281)	32.2 (18,194/56,494)
		2009	76.7 (17,500/22,813)	4.7 (1,079/22,813)	20.3 (1,079/5,313)	32.6 (22,813/69,979)

		2007	47.5 (13,865/29,165)	11.0 (3,221/29,165)	21.1 (3,221/15,300)	27.6 (29,165/105,849)
Total		2008	65.3 (21,232/32,511)	6.6 (2,139/32,511)	19.0 (2,139/11,279)	26.8 (32,511/121,395)
		2009	73.0 (27,330/37,437)	5.1 (1,917/37,437)	19.0 (1,917/10,107)	27.7 (37,437/135,093)

SET: single-embryo transfer.

ART: assisted reproductive technology.

IVF-ET: in vitro fertilization and embryo transfer.

ICSI: intracytoplasmic sperm injection.

For definitions of rates, see the text.

**Table 2:** Approximation formula with *R* ^2^.

Types of ART	Approximation formula		
	Linear approximation	Quadratic approximation	Exponential approximation
Fresh			
IVF-ET	*Y* = −0.25*X* + 22.78 (*R* ^2^ = 0.992)	*Y* = 0.0032*X* ^2^ − 0.61*X* + 31.98 (*R* ^2^ = 1)	*Y* = 39.704 exp ⁡ (−0.028*X*) (*R* ^2^ = 1)
*Y* (*X* = 100)	−2.04	3.79	2.41
ICSI	*Y* = −0.24*X* + 20.58 (*R* ^2^ = 0.996)	*Y* = 0.0026*X* ^2^ − 0.50*X* + 26.90 (*R* ^2^ = 1)	*Y* = 34.578 exp ⁡ (−0.028*X*) (*R* ^2^ = 1)
*Y* (*X* = 100)	−3.08	3.25	2.10
Subtotal	*Y* = −0.24*X* + 21.82 (*R* ^2^ = 0.992)	*Y* = 0.0032*X* ^2^ − 0.58*X* + 30.39 (*R* ^2^ = 1)	*Y* = 37.645 exp ⁡ (−0.028*X*) (*R* ^2^ = 1)
*Y* (*X* = 100)	−2.58	4.04	2.29

Frozen			
Frozen (intra-uterine)	*Y* = −0.25*X* + 23.60 (*R* ^2^ = 0.999)	*Y* = 0.0019*X* ^2^ − 0.49*X* + 31.51 (*R* ^2^ = 1)	*Y* = 68.083 exp ⁡(−0.035*X*) (*R* ^2^ = 0.998)
*Y* (*X* = 100)	−1.09	1.22	2.06
Subtotal	*Y* = −0.24*X* + 23.37 (*R* ^2^ = 0.999)	*Y* = 0.0020*X* ^2^ − 0.50*X* + 31.61 (*R* ^2^ = 1)	*Y* = 65.590 exp ⁡ (−0.034*X*) (*R* ^2^ = 0.998)
*Y* (*X* = 100)	−0.98	1.59	2.19

Grand total	*Y* = −0.24*X* + 22.20 (*R* ^2^ = 0.996)	*Y* = 0.0024*X* ^2^ − 0.52*X* + 30.52 (*R* ^2^ = 1)	*Y* = 46.148 exp ⁡ (−0.03*X*) (*R* ^2^ = 0.999)
*Y* (*X* = 100)	−1.40	2.03	2.30

ART: assisted reproductive technology.

IVF-ET: *in*

*vitro* fertilization and embryo transfer.

ICSI: intracytoplasmic sperm injection.

X: SET pregnancy rate. *Y*: multiple pregnancy rate.
